# Advancements in the application of artificial intelligence in the field of colorectal cancer

**DOI:** 10.3389/fonc.2025.1499223

**Published:** 2025-02-25

**Authors:** Mengying Zhu, Zhenzhu Zhai, Yue Wang, Fang Chen, Ruibin Liu, Xiaoquan Yang, Guohua Zhao

**Affiliations:** ^1^ Liaoning University of Traditional Chinese Medicine, Shenyang, China; ^2^ Department of General Surgery, Cancer Hospital of China Medical University, Liaoning Cancer Hospital & Institute, Shenyang, China; ^3^ Department of Gynecology, People’s Hospital of Liaoning Province, Shenyang, China

**Keywords:** colorectal cancer, artificial intelligence, diagnosis, treatment, prognosis prediction

## Abstract

Colorectal cancer (CRC) is a prevalent malignant tumor in the digestive system. As reported in the 2020 global cancer statistics, CRC accounted for more than 1.9 million new cases and 935,000 deaths, making it the third most common cancer worldwide in terms of incidence and the second leading cause of cancer-related deaths globally. This poses a significant threat to global public health. Early screening methods, such as fecal occult blood tests, colonoscopies, and imaging techniques, are crucial for detecting early lesions and enabling timely intervention before cancer becomes invasive. Early detection greatly enhances treatment possibilities, such as surgery, radiation therapy, and chemotherapy, with surgery being the main approach for treating early-stage CRC. In this context, artificial intelligence (AI) has shown immense potential in revolutionizing CRC management, serving as one of the most effective screening tools. AI, utilizing machine learning (ML) and deep learning (DL) algorithms, improves early detection, diagnosis, and treatment by processing large volumes of medical data, uncovering hidden patterns, and forecasting disease development. DL, a more advanced form of ML, simulates the brain’s processing power, enhancing the accuracy of tumor detection, differentiation, and prognosis predictions. These innovations offer the potential to revolutionize cancer care by boosting diagnostic accuracy, refining treatment approaches, and ultimately enhancing patient outcomes.

## 1 Introduction

CRC originates from malignant transformations in the mucosal epithelial cells of the colon or rectum and is one of the most prevalent malignancies within the digestive system (1). The 2020 global cancer statistics from the International Agency for Research on Cancer (IARC) of the World Health Organization reported more than 1.9 million new CRC cases and 935,000 deaths, making it the third most common cancer globally in terms of incidence and the second leading cause of cancer-related deaths (2). Early screening, diagnosis, and treatment can significantly extend the survival of CRC patients and improve their prognosis. Early screening primarily includes fecal occult blood tests, endoscopic examinations (such as colonoscopy), and imaging tests [such ascomputed tomography (CT) scans or magnetic resonance imaging (MRI)]. These screening methods can help detect early lesions of CRC, such as adenomatous polyps or precancerous changes, allowing for intervention before the cancer progresses to an invasive stage (3, 4). Once diagnosed with CRC, early diagnosis provides more treatment options, including surgical resection, radiation therapy, and chemotherapy (5). While traditional methods have proven effective, they do have certain limitations. For instance, colonoscopy, as outlined in the China Guideline for the Screening, Early Detection, and Early Treatment of CRC published in 2020 (6), may cause significant discomfort and pain for patients during the procedure. It can also lead to complications such as intestinal bleeding or perforation. Specifically, the incidence of bleeding during colonoscopy screening is reported to be 22.44 per 10,000 procedures (95% CI: 19.30–26.34). This method requires high technical proficiency from the physician, which limits its widespread use, particularly in resource-limited settings. Although colonoscopy is effective at detecting abnormalities, its invasiveness and the risk of missed diagnoses remain concerns. However, with the advancement of AI technology, there has been notable progress in AI applications in colonoscopy. For example, a real-time, robust AI diagnostic system for CRC developed by Yamada et al. (7) significantly reduces the risk of missing non-polypoid lesions. These innovations have the potential to improve screening accuracy, reduce missed diagnoses, and alleviate patient discomfort. In conclusion, AI technology has demonstrated its immense potential and strong advantages in the screening, diagnosis, and treatment of CRC.

AI is transforming modern healthcare by enhancing medical data analysis, image recognition, disease prediction, and personalized therapy (8). Using advanced algorithms and ML, AI processes large-scale medical data to uncover patterns that support early diagnosis and disease prevention (9). However, traditional ML algorithms are increasingly inadequate to meet the demands of complex research. DL, as an emerging research direction in AI, aims to simulate and understand how the human brain processes information. It represents a more sophisticated and powerful class of ML algorithms (10). Classic DL networks consist of input layers, hidden layers, and output layers, each composed of multiple nodes that process information. These nodes communicate and process information through simple yet nonlinear patterns, enabling DL networks to efficiently learn and extract features and patterns from complex data (11). DL has been extensively studied and reported for its applicability in cancer diagnosis and treatment. In the diagnostic phase, it efficiently detects and locates tumors by analyzing imaging and pathological data, and can even differentiate between different types of cancer. In terms of prognosis, DL algorithms utilize clinical and molecular data to predict patients’ survival and the risk of disease progression, providing a basis for personalized treatment plans (12). This technology has great potential to transform cancer care by improving diagnostic precision, refining treatment approaches, and enhancing patient outcomes.

## 2 Application of AI in CRC diagnosis

### 2.1 Colonoscopy

Endoscopic examination is the most sensitive method for CRC screening, allowing direct visualization of lesions and initial assessment of their characteristics using Narrow-Band Imaging (NBI) technology (13). Despite significant improvements in early tumor detection with advancements in technology, the risk of missed diagnoses persists, particularly for certain polyps and adenomas (14). In the context of rapid advancements in computer science, many researchers have begun exploring the use of AI for early detection and diagnosis of CRC. Masashi et al. (15) developed an AI-assisted CADe system using 73 annotated colonoscopy videos, segmented into 546 clips for ML evaluation. The clips were randomly split into two sets: one for training (learning samples) and the other for testing (evaluation of performance). The CADe system calculates a probability for polyp presence in each video frame, mimicking the confidence level of a human endoscopist. To achieve a system sensitivity greater than 90%, they conducted a Receiver Operating Characteristic (ROC) analysis, establishing a probability threshold of 15% and obtaining an area under the curve (AUC) of 0.87. To evaluate the performance of the CADe system, sensitivity, specificity, and accuracy were calculated for each frame. A detection was considered positive if the probability surpassed the threshold. Sensitivity was determined by dividing the number of correctly identified frames by the total number of polyp frames in the test set. The results revealed that the system achieved 90.0% sensitivity, 63.3% specificity, and 76.5% accuracy at the frame level. In a polyp-based analysis, the system detected 94% of the polyps (47/50), with a false positive rate of 60% (51/85). A high false positive rate may lead to unnecessary tests and treatments, increasing patient discomfort, anxiety, and medical costs, while also exacerbating the strain on medical resources. Kudo et al. (16) evaluated the performance of the AI system EndoBRAIN in distinguishing between tumor and non-tumor lesions in colonoscopy images. The system was trained using 69,142 endoscopic images, and its diagnostic performance was compared with that of 30 endoscopists, including 20 trainees and 10 experts. In the analysis of chromoendoscopic images, EndoBRAIN reached a sensitivity of 96.9%, specificity of 100%, and accuracy of 98%. For NBI, the system achieved a sensitivity of 96.9%, specificity of 94.3%, and accuracy of 96.0%. Overall, EndoBRAIN’s performance was significantly better than that of trainees and comparable to that of experts in both chromoendoscopic and NBI. Jin et al. (17) created a convolutional neural network (CNN) to assess small colorectal polyps. The network was trained on images of 1100 adenomatous and 1050 hyperplastic polyps sourced from 1379 patients, and it was tested on 300 images. The CNN demonstrated an accuracy of 86.7% in differentiating adenomatous polyps from hyperplastic ones. Compared to the CNN, 22 endoscopists’ accuracy improved from 82.5% to 88.5%, with novice endoscopists’ accuracy increasing to 85.6%. The CNN also significantly reduced the diagnostic time from 3.92 seconds to 3.37 seconds (P = 0.042). These results demonstrate that the CNN significantly enhances the accuracy and efficiency of novice endoscopists, reducing reliance on skill levels. In summary, the use of AI in colonoscopy is proving to be a valuable advancement in CRC screening. AI systems, such as CADe, EndoBRAIN, and CNNs, have demonstrated the ability to improve the detection of polyps and lesions with high sensitivity and accuracy. These systems not only support experienced endoscopists but also assist novice practitioners by reducing diagnostic time and increasing overall detection rates. As AI technology advances, its incorporation into clinical practice could improve early detection, minimize missed diagnoses, and ultimately lead to better patient outcomes in CRC screening.

### 2.2 Pathological diagnosis

Pathological diagnosis is regarded as the “gold standard” in cancer diagnosis because it accurately determines tumor type and stage by examining cells and tissues under a microscope. However, this standard relies on traditional microscopy techniques, which result in inefficiencies and limitations in information processing. To enhance the efficiency and accuracy of pathology, the integration of digital pathology and AI is increasingly recognized as a crucial advancement. Väyrynen et al. (18) conducted a computational analysis of H&E-stained slides to identify various immune cells in CRC and assessed their impact on survival using multivariable Cox regression. The results revealed that high densities of stromal lymphocytes and eosinophils were associated with improved cancer-specific survival, with these findings validated in an independent cohort. Additionally, GTumor: Immune cellfunction analysis further confirmed the association between high immune cell densities and better prognosis. These findings demonstrate the potential of ML in assessing immune cells in H&E-stained slides for precision medicine. Analyzing glandular morphology in colorectal pathological images is essential for CRC grading; however, manual segmentation is labor-intensive and prone to variability between observers. To address these challenges, Graham et al. (19) designed a fully CNN that minimizes information loss by reintegrating the original image at various stages and utilizing dilated spatial pyramid pooling to preserve resolution. They incorporated uncertainty through random transformations and generated uncertainty maps to boost segmentation and prediction accuracy. Their approach outperformed all other methods on the 2015 MICCAI GlaS Challenge dataset, achieving state-of-the-art results. Moreover, they introduced MILD-Net+ for the simultaneous segmentation of glands and lumens, further improving diagnostic performance. Kiehl et al. (20) trained a CNN using histological whole-slide images from the DACHS cohort to predict lymph node metastasis (LNM) in CRC. They combined this with clinical data for logistic regression analysis. In the internal test set, slide-based AI predictor (SBAIP) achieved an AUROC of 71.0%, which improved to 74.1% when combined with the clinical classifier. However, in the external TCGA test set, SBAIP’s AUROC decreased to 61.2%. This suggests that while DL image analysis combined with clinical data can aid in predicting LNM, improving SBAIP’s performance on external data is necessary. These advancements highlight the revolutionary potential of integrating digital pathology and AI in cancer diagnosis. By improving diagnostic accuracy, efficiency, and speed, these innovations offer new pathways for achieving more precise and timely patient care in oncology.

### 2.3 CRC staging diagnosis

Currently, imaging methods for staging CRC in clinical practice mainly include CT, MRI, and endorectal ultrasound (ERUS) (21). MRI is the preferred imaging method for rectal cancer (RC)due to its ability to clearly assess tumor location, depth, LNM, and invasion of surrounding organs (22). While MRI provides detailed images, its diagnostic accuracy depends on the physician’s experience and can vary. In contrast, AI enhances diagnostic accuracy and consistency by analyzing extensive image data and detecting subtle changes. AI reduces human error, improves staging efficiency, and supports more objective treatment decisions, ultimately benefiting patient outcomes. Shu et al. (23) analyzed data from 317 patients using various ML algorithms to predict preoperative extramural venous invasion (EMVI) in rectal cancer via multiparametric MRI radiomics. Among the algorithms, the Bayesian model showed good performance with an AUC of 0.744 (training set) and 0.738 (test set). However, the best results were achieved by a combined model using clinical and imaging features, which had an AUC of 0.839 (training set) and 0.835 (test set), indicating excellent diagnostic potential for individualized EMVI prediction. Zhao et al. (24) created a radiomics nomogram using relaxation imaging to predict EMVI in rectal cancer (RC). The study involved 94 RC patients who had surgery, with 65 patients used for training and 29 for validation. Feature selection was carried out using the Least Absolute Shrinkage and Selection Operator (LASSO), and the nomogram was developed through multivariable logistic regression. The radiomics model achieved areas under the ROC curve (AUC) of 0.912 and 0.877 for the training and validation groups, respectively, while the nomogram showed AUCs of 0.925 and 0.899. The model outperformed radiologists’ subjective assessments in terms of diagnostic performance. Jia and his team’s study assessed a nomogram combining IVIM-DWI and radiomics for preoperative identification of non-enlarged lymph node metastases (N-LNM) in 126 rectal adenocarcinoma patients. The model, which used measures of ADC, D, D*, and f, showed that the LN+ group had lower D* and higher f values compared to the LN- group. It performed well in predicting N-LNM, with an AUC of 0.864 in the training cohort (25). These studies indicate that the combination of AI and radiomics significantly enhances the accuracy and reliability of staging CRC. By integrating clinical and imaging data, advanced predictive models not only optimize the diagnostic process but also provide more precise guidance for personalized treatment.

## 3 Application of AI in the treatment of CRC

### 3.1 Surgical therapy

Surgical treatment is considered the primary and most effective method for managing patients with CRC. Because it has the potential to completely remove the tumor and, in many cases, offer a cure (26). Laparoscopic surgery has become a primary method for treating CRC, accounting for more than half of all CRC surgeries (27). Despite benefits like reduced trauma and faster recovery, laparoscopic surgery has notable limitations. Traditional systems may suffer from unstable camera support, affecting image quality and field of view. Two-dimensional imaging limits depth perception, and equipment mobility constraints can impact surgical flexibility. Additionally, the ergonomic design often neglects surgeon comfort, leading to fatigue during long procedures (28). With the advancement of AI, CRC surgery has entered a new era, exemplified by the significant progress made with the da Vinci Surgical System. The da Vinci Surgical System is an advanced robotic-assisted surgery platform that significantly enhances the safety and effectiveness of surgeries by providing higher precision and better visualization. This system allows surgeons to perform operations using minimally invasive techniques, through several small incisions rather than traditional large ones. This approach not only reduces postoperative pain and recovery time but also lowers the risk of complications (29, 30). Kim et al. (31) evaluated the safety and performance of the da Vinci SP^®^ surgical system in 50 colorectal surgery patients. The study found that with increasing surgical experience, operation times significantly decreased, all surgeries were successfully completed, with only 6 minor adverse events reported within 3 months post-surgery, and no local recurrences within 1 year, with only 1 case of systemic recurrence. Jung et al. (32) evaluated short-term outcomes of robotic-assisted colon cancer surgeries using the da Vinci SP and Xi systems at two tertiary centers from November 2020 to December 2022. Patients using the SP system had shorter incision lengths (5.0 cm vs. 9.4 cm), lower pain scores at 8 hours (3.0 vs. 3.5) and 24 hours post-operation (2.9 vs. 3.3), and shorter hospital stays (5 days vs. 6 days). Postoperative complication rates were similar (SP: 7.5% vs. Xi: 13.2%). The da Vinci SP system showed benefits in cosmesis, pain, and recovery duration compared to the Xi system. This robotic-assisted technology offers promising improvements in both precision and surgeon comfort. In conclusion, while traditional laparoscopic surgery remains a primary treatment for CRC, robotic-assisted systems like the da Vinci Surgical System offer significant advantages. These systems enhance precision, visualization, and surgeon comfort, leading to reduced trauma, faster recovery, and lower complication rates.

### 3.2 Radiotherapy

Radiotherapy is the preferred neoadjuvant treatment for intermediate and locally advanced RC because it effectively reduces tumor size, alleviates local burden, increases surgical success rates, lowers the risk of recurrence, and improves patient quality of life (33, 34). Magnetic Resonance-Guided Radiation Therapy (MRgRT) is a major advancement in radiation therapy, using real-time MRI for precise tumor and organ visualization. MRgRT allows doctors to monitor tumors and surrounding tissues dynamically, improving treatment accuracy and personalization while minimizing damage to healthy tissues. It is particularly effective for complex soft tissue tumors, offering superior image resolution and localization compared to traditional imaging-guided radiation therapy (35). Ferrari et al. created an AI model utilizing high-resolution T2-weighted MRI texture analysis to predict pathological complete response (CR) and identify non-responders (NR) in patients with locally advanced rectal cancer (LARC). The study involved 55 patients who underwent MRI during chemoradiotherapy, with histopathology as the reference. A random forest classifier, trained on 28 patients, achieved average AUCs of 0.86 for CR and 0.83 for NR in a validation cohort of 27 patients, surpassing the performance of standard care (36). Research shows that online adaptive radiotherapy (ART) can dynamically adjust treatment plans using real-time imaging information, effectively reducing radiation therapy side effects for RC patients by optimizing target areas and minimizing unnecessary radiation exposure (37). Jong et al. (38) studied cone-beam CT (CBCT) for online ART in 12 rectal cancer patients receiving preoperative 5 × 5 Gy radiotherapy. They used a 5 mm PTV margin (8 mm for head and tail) and integrated software for planning and adjustments. Average treatment time was 34 minutes, with 20 minutes for adjustments, and manual target volume adjustments were needed in 50% of cases. Results indicated excellent plan quality, target coverage, and patient compliance, confirming the method’s clinical feasibility. In summary, radiotherapy plays a crucial role in the treatment of intermediate and locally advanced rectal cancer, especially when combined with MRgRT and online ART, which significantly improve treatment accuracy and personalization. AI, particularly in the application of MRI texture analysis, aids in predicting treatment response and optimizing treatment plans, further enhancing the outcomes and prognosis of rectal cancer patients. The integration of these innovative technologies is driving the precision and intelligence of radiotherapy treatment forward.

## 4 Application of AI in prognostic prediction for CRC

Traditional prognostic methods for CRC primarily rely on clinical data and pathological indicators, such as tumor staging, grading, and biomarkers. While these methods can provide some prognostic information, they are limited by subjective judgment and the finite nature of data samples, which results in certain constraints (39, 40). In recent years, the rise of AI has provided new solutions to these issues. Through DL and data mining techniques, AI can process large amounts of complex clinical data and uncover potential prognostic information, leading to more precise predictions. Zhao et al. (41) developed a DL model that can automatically quantify the tumor-stroma ratio (TSR) in CRC from HE-stained whole slide images (WSI). Using CNNs and transfer learning, the model segments WSIs and computes TSR. In two test cohorts (discovery cohort N=499, validation cohort N=315), high TSR was associated with lower overall survival (OS). Integrating TSR with other risk factors in the Cox model demonstrated improved prognostic capability. Kather et al. (42) investigated the use of deep CNNs in extracting prognostic factors for CRC. They trained a CNN using 86 tissue samples and over 100,000 HE image patches, achieving an accuracy of over 94%. Using this tool, they analyzed 862 HE slices from 500 CRC patients and calculated a “deep stroma score,” which was found to be an independent prognostic factor for OS (HR 1.99 [1.27-3.12], p = 0.0028). This finding was also validated in the DACHS independent cohort, showing that the score has independent prognostic significance for OS, CRC-specific OS, and RFS (p < 0.01). This indicates that CNNs can effectively assess the tumor microenvironment and predict prognosis, although further validation is needed before clinical application. In conclusion, AI, particularly DL techniques, has shown great promise in overcoming the limitations of traditional prognostic methods in CRC. By processing large and complex clinical datasets, AI can identify key prognostic factors, such as the TSR and deep stroma scores, which provide more accurate and reliable predictions of patient outcomes. These advancements highlight the potential of AI in improving the precision of CRC prognosis, offering significant clinical value in personalized treatment planning and patient management.

## 5 Summary and outlook

AI, particularly DL, has greatly advanced CRC diagnosis and treatment by revolutionizing medical data analysis. AI algorithms uncover key patterns for early CRC screening, enhancing accuracy and enabling precision diagnostics and personalized treatment strategies. For example, AI analysis of pathological images can detect abnormalities earlier and make more precise diagnoses, leading to more personalized treatment plans. These advancements, however, come with challenges and limitations. First and foremost, the cost-effectiveness of AI implementation must be considered. During the initial development phase, training AI models requires large amounts of high-quality data, the collection and processing of which often incur significant expenses. Additionally, the applicability and generalizability of AI models across different populations is a critical issue. While many AI models perform excellently on specific training sets, differences in demographics and environments may affect their performance. Furthermore, the source and diversity of training data are crucial to the external validity of AI models. If an AI model is trained solely in a particular region or population without accounting for global diversity, its widespread application may be limited. Moreover, in resource-poor settings, there may be insufficient funds to acquire and maintain high-end AI equipment, and training healthcare professionals to use these systems requires both time and resources. Data privacy and security concerns may also pose significant barriers in certain regions. To address these challenges, future research should focus on several key areas: constructing more diverse and high-quality training datasets to ensure that AI systems can better adapt to the diagnostic needs of different populations worldwide; optimizing AI algorithms and hardware to reduce reliance on expensive equipment; developing more reliable validation methods to ensure the effectiveness and safety of models in various clinical environments; ensuring that patient data protection complies with international privacy and security regulations to prevent misuse or leakage of personal information; enhancing physician training to ensure they can effectively use AI-assisted diagnostic tools; and fostering greater understanding and trust in AI technology among patients. In the future, AI can provide more accurate diagnoses, optimize treatment plans, and offer better treatment outcomes and higher survival rates for patients. Continued advancement in AI research and application will contribute to more personalized and precise medical services, ultimately improving patients’ overall health and quality of life.
